# A new protein linear motif benchmark for multiple sequence alignment software

**DOI:** 10.1186/1471-2105-9-213

**Published:** 2008-04-25

**Authors:** Emmanuel Perrodou, Claudia Chica, Olivier Poch, Toby J Gibson, Julie D Thompson

**Affiliations:** 1 Institut de Génétique et de Biologie Moléculaire et Cellulaire (IGBMC), Department of Structural Biology and Genomics, F-67400 Illkirch, France; 2 Institut National de la Santé et de la Recherche Médicale (INSERM), U596, F-67400 Illkirch, France; 3 The Centre National de la Recherche Scientifique (CNRS), UMR7104, F-67400 Illkirch, France; Université Louis Pasteur, F-67000 Strasbourg, France; 4 European Molecular Biology Laboratory, Meyerhofstraße 1, 69012 Heidelberg, Germany

## Abstract

**Background:**

Linear motifs (LMs) are abundant short regulatory sites used for modulating the functions of many eukaryotic proteins. They play important roles in post-translational modification, cell compartment targeting, docking sites for regulatory complex assembly and protein processing and cleavage. Methods for LM detection are now being developed that are strongly dependent on scores for motif conservation in homologous proteins. However, most LMs are found in natively disordered polypeptide segments that evolve rapidly, unhindered by structural constraints on the sequence. These regions of modular proteins are difficult to align using classical multiple sequence alignment programs that are specifically optimised to align the globular domains. As a consequence, poor motif alignment quality is hindering efforts to detect new LMs.

**Results:**

We have developed a new benchmark, as part of the BAliBASE suite, designed to assess the ability of standard multiple alignment methods to detect and align LMs. The reference alignments are organised into different test sets representing real alignment problems and contain examples of experimentally verified functional motifs, extracted from the Eukaryotic Linear Motif (ELM) database. The benchmark has been used to evaluate and compare a number of multiple alignment programs. With distantly related proteins, the worst alignment program correctly aligns 48% of LMs compared to 73% for the best program. However, the performance of all the programs is adversely affected by the introduction of other sequences containing false positive motifs. The ranking of the alignment programs based on LM alignment quality is similar to that observed when considering full-length protein alignments, however little correlation was observed between LM and overall alignment quality for individual alignment test cases.

**Conclusion:**

We have shown that none of the programs currently available is capable of reliably aligning LMs in distantly related sequences and we have highlighted a number of specific problems. The results of the tests suggest possible ways to improve program accuracy for difficult, divergent sequences.

## Background

Many eukaryotic proteins have highly modular architectures. Multidomain proteins are usual for transmembrane receptors, signalling proteins, cytoskeletal proteins, chromatin proteins, transcription factors and so forth. As a consequence, many programs have been developed for the detection and alignment of protein domains. Online resources can now provide a good overview of the globular domain architecture of a polypeptide sequence and the functions these domains are likely to perform, e.g. Pfam [1], SMART [2], Interpro [3]. However, less research has been directed towards the analysis of the large segments of multidomain proteins that are non-globular, intrinsically lacking the capability to fold into a defined tertiary structure [4,5]. Sometimes such regions may simply act as linkers connecting globular domains and in this case, the sequence of amino acids is not critical to function. Very often, however, these unstructured regions contain important functional sites such as protein interaction sites, cell compartment targeting signals, post-translational modification sites or cleavage sites. Large parts of many proteins, such as the insulin receptor substrates, or sometimes even the entire protein, such as the Alzheimer's protein Tau [6], are natively unstructured. The functional sites within these unstructured regions can often be defined as short, linear motifs (LMs) – linear in the sense that only the local peptide sequence is relevant to function. In order to avoid confusion, in this paper we will use the term 'sequence' to refer to the full-length protein, while a specific region of a protein sequence will be referred to as a 'segment' or a 'motif'. The Eukaryotic Linear Motif resource (ELM) has entries describing ~130 varieties of linear motif [7], but it is not fully comprehensive with respect to current literature and it has been estimated that hundreds more have yet to be discovered [8]. When eubacterial, archaebacterial and viral motifs are also considered, the true number of unknown functionally important LMs is likely to be huge. Given the fundamental roles these motifs play in cell regulation and signalling, identifying these motifs will be of crucial importance in many biological disciplines.

Until recently, and in stark contrast to protein domain discovery, the bioinformatics field has had a negligible impact on LM discovery: motif discovery is generally performed by low throughput experimental delineation of protein interaction segments. The central problem confounding computational methods has been the lack of significance of motif matches when searching sequence databases, making it impossible to confidently identify all the motifs present in a given protein sequence by simple sequence analysis tools. The majority of LMs are between 3 and 10 amino acids in length and most have one or more ambiguous (variable) or wildcard (totally variable) residues. Their short and degenerate nature makes real LMs difficult to distinguish from the background distribution of randomly occurring false positive motifs. Nevertheless, efforts are now underway to develop bioinformatics tools that will contribute to the linear motif discovery problem. As a first step, it is necessary to catalogue linear motifs and particular instances that are known to be functional. Such data collections include the eukaryotic linear motif (ELM) resource [7] and ScanSite [9]. A number of tools have been developed, e.g. ELM [7], QuasiMotiFinder [10], MiniMotif [11] and the ACS method [12], that employ various methods, such as domain masking and evolutionary filtering respectively, to discover new occurrences of previously known motifs. Other methods, such as the LMD method [8] (implemented in the web server DILIMOT [13]), SLiMDisc [14], SLiMFinder [15] and Miner [16], explicitly attempt novel LM discovery using large scale interaction datasets and/or motif conservation.

One of the major limitations in predicting short linear motifs is the evaluation of the many potential motifs found in each protein, to distinguish between true functional sites and incorrect occurrences of a given pattern. In the worst case, there are motifs which have such low support and low information content as to be almost indistinguishable from random noise in most datasets, e.g. the PCSK cleavage site K/RR [17] which plays a role in proteolytic processing of neuropeptide and peptide hormone precursors, or the peroxisomal targeting motif WxxxY/F (where x represents any arbitrary amino acid) [18]. It is vitally important, therefore, to develop novel scoring methods or to consider other information, such as contextual information, e.g. loop region, N/C-terminus, cellular localisation, if such data is available, or evolutionary information, since motifs conserved during evolution are more likely to be functional. Conservation has been shown to be an essential factor in the prediction of functional motifs. For example, many motif discovery systems, such as LMD, QuasiMotiFinder Miner, MiniMotif and the ACS method use a combination of traditional motif scores and evolutionary conservation to rank potential motifs. It is worth pointing out though, that while LMD explicitly utilises conservation, the method used is alignment-free and, as such, would not be affected by the developments described in this article. SLiMFinder and SLiMDisc make use of automated multiple alignments and conservation scores to help visualise and interpret results. We have also recently developed a rapid automated conservation scoring pipeline suitable for real time operation in the ELM resource [19].

It follows therefore that, in order to exploit evolutionary information optimally, we need to construct multiple sequence alignments of the highest quality. LMs that occur in several different phyla should appear as short patches of conservation in this alignment. However, a large majority of LMs are found in the natively disordered regions [20] that are difficult to align using classical multiple sequence alignment programs, which are better adapted to protein domain alignments. The biological relevance of the alignments produced by these programs is usually assessed by systematic comparison with established benchmark sets, e.g. BAliBASE [21], Prefab [22] or Sabmark [23], based on 3D structure superpositions of globular domains. The introduction of these objective benchmarks has had a considerable effect on the evolution of alignment algorithms and has led to a significant improvement in overall multiple alignment quality [24]. However, there is also a risk that alignment software optimised on structure superpositions has been overfitted to globular domains and may not adequately account for awkward features of full length protein sequences, such as N- and C-terminal extensions and motif-rich non-globular sequence segments. Therefore, to evaluate the ability of multiple alignment methods to identify and align LMs, new test sets are now needed. Benchmarks have already been developed for motif discovery in genomic DNA sequences, such as transcription factor binding sites, e.g. [25], but these benchmarks are not generally organised into evolutionarily related sets that might be used to evaluate multiple sequence alignment programs. Another reference database, IRMBASE [26], consists of simulated conserved motifs implanted into non-related artificial protein sequences. However, this benchmark does not reflect the problems associated with identifying and aligning the short linear motifs that are essential for the function of real multimodular proteins.

The main objective of the work presented here is to provide a standard way to assess the ability of a multiple alignment program to correctly align the linear motifs occurring in a set of related sequences. However, if the multiple alignment is to be used in a subsequent motif discovery system, it is important that (i) the sequences containing the motif should be accurately aligned and (ii) the sequences that do not contain the motif should not be aligned in the corresponding region. To address these issues, we have developed a new Reference Set that has been incorporated in the BAliBASE benchmark suite [21]. The benchmark includes example multiple alignments for most of the motifs annotated in the ELM resource [7]. For each LM, a representative set of homologous sequences has been selected and a multiple alignment of the complete sequences (MACS) has been constructed and manually refined. A number of different test subsets are provided, representing typical scenarios and problems that occur when trying to align the motifs in the context of a global multiple alignment.

Using the new BAliBASE Reference Set, we then evaluated the accuracy of the motif alignments obtained from a number of widely used or recently developed multiple alignment programs. The performance of the different programs was assessed by comparing the alignments constructed by each program with the reference alignments. We show that none of the programs currently available is capable of reliably aligning LMs in distantly related sequences and we highlight a number of specific problems. This will hopefully generate interest in developing new algorithms and should provide program developers with guidelines for future enhancements that will improve the quality of motif alignments.

## Methods

**Figure 1:**
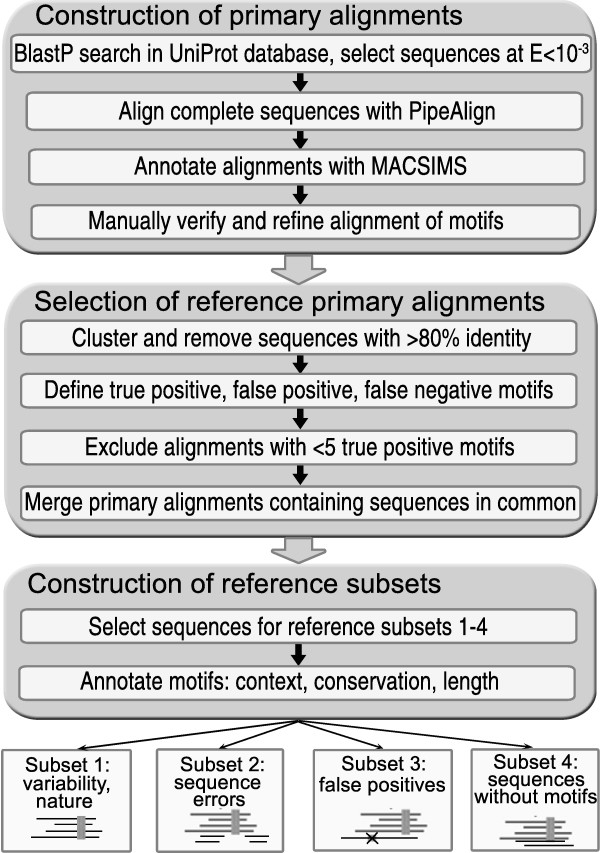
**BAliBASE Reference Set 9 construction protocol**. Flow-chart showing the 3 major steps of the protocol used to construct the BAliBASE Reference Set 9.

The new Reference Set in the BAliBASE benchmark contains multiple alignments of sequences with functional linear motifs (LMs). To avoid confusion, the term 'sequence' will be used to refer to the full-length protein, while a specific region of a protein sequence will be referred to as a 'segment' or a 'motif'. The alignments are constructed from a set of reference sequences with experimentally verified motifs, known as ELM motif instances, extracted from the ELM database [7]. Putative functional sites in ELM are identified by patterns (regular expressions) that are similar to PROSITE patterns [27]. Although the ELM regular expressions are highly discriminative and provide a useful resource for the identification of functional LMs, these regular expressions are not 100% accurate and may either (i) exclude true motifs or (ii) may find false, but apparently plausible, motif matches [7]. For this study, the 406 motif instances in the ELM database associated with experimental evidence were extracted. They correspond to the 123 different linear motifs (different regular expressions) present in the ELM version I-2007. For each reference sequence containing an ELM motif instance, a semi-automatic protocol has been developed to detect related sequences in the UniProt [28] protein database, and to construct a 'primary' multiple alignment that is then used to select suitable sequences for each subset in the Reference Set. A general overview of the protocol is shown in the flowchart in figure 1.

### Primary multiple alignments

For each reference sequence containing an ELM motif instance, the first step involves the detection of similar sequences in the Uniprot database [28] and the construction of a multiple alignment of the complete sequences, using the following protocol:

(i) The Uniprot database is searched using the BlastP program [29] with the full-length reference sequence as query and all sequences detected with E < 10^-3 ^are initially selected.

(ii) The selected full-length sequences are aligned using the PipeAlign [30] protein family analysis toolkit to construct a Multiple Alignment of Complete Sequences (MACS). At this stage, sequences that do not contain any regions considered to be homologous to the reference sequence are removed from the MACS, using the LEON program [31].

(iii) The sequences in the MACS are then annotated using the MACSIMS information management system [32]. MACSIMS mines information from the public databases, including known structural domains, functional sites and other motifs. At the same time, a number of prediction algorithms are run to detect sequence features, including transmembrane regions, low-complexity segments and disordered regions. At this stage, all motifs that match the ELM regular expressions are also identified and annotated using a Perl script.

(iv) Finally, the automatic multiple alignment is manually verified and refined to assure the accurate alignment of the potential motifs with the current motif instance. A number of factors were taken into account during the manual validation, including the MACSIMS functional annotations, the presence of known globular domains or functional sites or the presence of conserved sequence segments close to the instance LM. The globular domains were generally well aligned by the PipeAlign toolkit and the manual refinement was limited to the potential LMs, identified by the ELM regular expressions. Low complexity regions were not realigned, as these could not be validated in most cases. During the manual refinement, any sequences containing motifs that could not be validated unambiguously were removed from the multiple alignment. These included motifs occurring in unrelated proteins (i.e. sharing no globular domains with the query sequence) and sequences with multiple occurrences of a motif, where the correspondence with the ELM motif instance was uncertain.

### Selection of reference primary alignments

**Figure 2:**
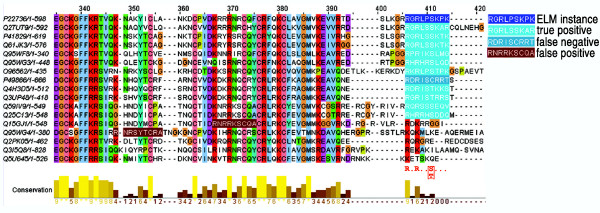
**Example alignment in BAliBASE Reference Set 9**. Part of an alignment, showing the MOD_PKM_1 ELM regular expression (R.R.. [ST]...), with examples of true positive, false positive and false negative motifs. The last three sequences do not contain any examples of the motif and cannot be aligned unambiguously in the region of the motif instance.

Each Primary multiple alignment contains the complete set of sequences detected by BlastP, that share some similarity with the query sequence. In order to construct benchmark test sets, sequences are selected that correspond to a certain number of criteria. The following protocol is used to identify suitable alignments and sequences:

(i) In order to reduce the size of the test sets, redundant sequences sharing >80% residue identity that are easy to align, are removed in a clustering step, based on the multiple sequence alignment.

(ii) True positive, false positive and false negative motifs are defined (see figure 2). A true positive motif corresponds to a motif that matches the ELM regular expression, and that occurs in a region that can be aligned on the motif instance. A false positive motif is a motif that matches the ELM regular expression, but that is detected in a region that does not align on the motif instance. In contrast, a false negative motif is defined as a sequence segment that can be aligned unambiguously in the region of the motif instance, but that does not match the ELM regular expression. In the example shown in figure 2, the motif in P49866 is defined as a 'false negative', since it can be aligned unambiguously with other sequences (Q4H3D5 and Q3UP48) that do match the ELM regular expression. Sequences that do not contain any matches to the ELM regular expression and that cannot be aligned manually in the region of the motif instance are also included in the primary alignments. It should be noted that this classification of true positive, false positive and false negative motifs refers to the current motif instance only, based on the presence of a match to the ELM regular expression and the quality of the alignment in this region. The classification is useful for the purposes of this study, but it does not imply anything about the alignment of the motifs at other positions, nor does it imply anything about the functionality of the motifs, which cannot be assessed exclusively by sequence analysis tools. The false positive motifs provide a control to avoid over-optimisation of multiple alignment algorithms when using the Reference Set as a benchmark, since aligning the false positive matches on the ELM motif instance will lead to a misalignment of the globular regions in the sequences (see examples in figure 2). The false negative motifs are important because, given their amino acid sequence they should be aligned on the motif instance, even if they do not match the ELM regular expression.

(iii) Primary alignments containing less than 5 sequences with true positive motifs were excluded from the benchmark.

(iv) Finally, primary alignments with at least one sequence in common were merged together to form a single reference alignment. This step is necessary because the original set of query sequences contains a certain number of homologous sequences, resulting in an overlap between the corresponding sets of BlastP detected sequences.

### Construction of reference subsets

The reference primary alignments are then divided into 4 Subsets, designed to evaluate the accuracy of multiple alignment algorithms under different conditions. Subsets 1–4 are constructed by automatically selecting the sequences from the Primary multiple alignment that correspond to a number of specific criteria.

(i) For Subset 1, only sequences with validated LMs (true positive or false negative motifs) are selected. Additional "false positive" motifs that match the ELM regular expression may be present at other positions in the alignment, as long as the LM corresponding to the motif instance can be identified unambiguously. The subset is further organised into three different groups, according to sequence variability: <20% identity, 20–40% identity and 40–80% identity.

(ii) Subsets 2–4 include the sequences in Subset 1, together with additional sequences that cannot be aligned in the region of the motif instance. These additional sequences may cause problems for automatic multiple alignment programs:-

▪ Subset 2 includes additional sequences that share some homology with the reference sequence, but that have possible 'errors' (badly predicted sequences, fragments, splicing variants) in the region of the motif instance. Although these 'error' sequences do not contain a validated LM, they may or may not contain false positive matches to the ELM regular expression elsewhere in the sequence.

▪ Subset 3 includes additional sequences with false positive matches to the ELM regular expression, that are found at other positions in the alignment and do not correspond to the motif instance.

▪ Subset 4 includes additional sequences that do not have any examples of the LM, either in the region of the motif instance or elsewhere in the alignment.

(iii) For each alignment in the benchmark, the validated LMs aligned on the motif instance are characterised according to three different features:

• Structural context (globular versus disordered regions). Globular segments were predicted using the IUPred program [33]. IUPred estimates the capacity of polypeptides to form stabilizing contacts. The underlying assumption is that globular proteins make a large number of interresidue interactions, providing the stabilizing energy to overcome the entropy loss during folding. In contrast, disordered regions are assumed to have special sequences that do not have the capacity to form sufficient interresidue interactions.

• Motif conservation. Motifs are defined as 'conserved' if all the true positive sequences in the multiple alignment share at least one conserved residue. Motifs in which no residues are conserved are defined as 'variable' motifs. The conservation is calculated based only on the validated motifs present in the multiple alignment, and does not necessarily correspond to non-wildcard positions in the motif regular expression.

• Motif length (number of residues in the motif instance, including wildcard positions).

### Definition of a quality score for alignment of motifs

To assess the accuracy of the programs evaluated in this study, we calculate a sum-of-pairs score (SPS) which estimates the accuracy of the alignment of the motifs. The SPS corresponds to the percentage of correctly aligned pairs of residues in the alignment produced by the program. Only the sequences with true positive or false negative motifs, which can be aligned unambiguously in the region of the reference motif, are taken into account. The alignment of the other sequences has no effect on the SPS quality score.

Suppose we have a test alignment and that N sequences in the alignment contain true positive or false negative motifs. Suppose also that the reference motif corresponds to M columns in the alignment. We can designate the i^th ^column in the alignment of the motif by A_i1_, A_i2_,...... A_iN_. For each pair of residues A_ij _and A_ik_, we then define p_ijk _such that p_ijk _= 1 if residues A_ij _and A_ik _are aligned with each other in the reference alignment, otherwise p_ijk _= 0. The score S_i _for the i^th ^column is defined as:

$$S_{i}=\sum_{j=1,j\neq k}^{N}\sum_{k=1}^{N}p_{ijk}$$

The SPS for the alignment is then:

$$SPS=\frac{\sum_{i=1}^{M}S_{i}}{\sum_{i=1}^{M_{r}}S_{ri}}$$

where M_r _is the number of columns corresponding to the reference motif and S_ri _is the score S_i _for the i^th ^column in the reference alignment. The score thus includes all columns in the alignment that correspond to residues in the motif instance, including variable and wildcard positions. For variable length motifs, positions containing gaps in the reference alignment are ignored.

### Multiple alignment programs tested

Eight different alignment programs were tested, representing the mostly widely used approaches for the construction of multiple alignments:

1. ClustalW [34] (version 1.83) performs a progressive multiple alignment using a series of pairwise alignments that follow the branching order in a phylogenetic tree. A global dynamic programming algorithm is used to construct an alignment of the full length of the sequences.

2. Dialign [35] (version 2.2.1) uses an alternative 'segment-to-segment' alignment method. Segments consisting of locally conserved residue patterns or motifs, rather than individual residues, are detected and then combined to construct a local multiple alignment of the most conserved regions of the sequences.

3. T-Coffee [36] (version 5.05) uses information from a pre-compiled library of different pairwise alignments including both local and global alignments, which are then incorporated in a global multiple alignment.

4. Mafft [37] (version 6.24) is an efficient method that includes fast pairwise alignments, and a progressive multiple alignment strategy that uses local anchors to reduce the area of the dynamic programming matrix that must be computed. Here, we have tested two alternative strategies: Mafft_fftns2 (using a fast Fourier transform at the pairwise alignment stage and no iterative refinement) and Mafft_linsi (using a local dynamic programming algorithm to construct more accurate pairwise alignments and an iterative refinement method using weighted sum-of-pairs and consistency scores).

5. Muscle [24] (version 3.6) uses a similar strategy to that developed in Mafft and includes a variety of options that offer different trade-offs between speed and accuracy. Muscle_fast uses k-mer counting to estimate the pairwise sequence distances and identifies diagonals that are used as local anchors for the progressive alignment, while the default Muscle program uses more accurate progressive alignment options and includes an iterative refinement strategy.

6. Probcons [38] (version 1.12) uses HMM-derived posterior probabilities and three-way alignment consistency in a global, progressive alignment, together with an iterative refinement step.

7. Kalign [39] (version 2) again follows a strategy analogous to the standard progressive method, but uses the Wu-Manber approximate string-matching algorithm in the sequence distance calculation.

8. Mummals [40] (version 1.01) incorporates more complex pairwise alignments based on Hidden Markov Models, and a probabilistic consistency-based progressive multiple sequence alignment.

All the programs were installed on a Sun Enterprise V40z server running RedHat Enterprise Linux 4 and each program was tested using default parameters and, for the purposes of this study, no optimisation of parameters such as residue weight matrix or gap penalties was performed.

### Availability

All the reference alignments are available for viewing on the WWW at  and are also provided in MSF format. The annotated alignments are also provided in XML format. A C program is provided to estimate the accuracy of the multiple alignments constructed by different alignment programs compared to the BAliBASE references. The complete database and the evaluation program are available for downloading by ftp from .

## Results

The ELM database [7] provides a unique source of eukaryotic sequences with experimentally verified instances of functional motifs. These functional sites are identified by patterns (ELM regular expressions) that are similar to PROSITE patterns [27]. The sequences containing the functional motifs, known as ELM motif instances, were used to search the protein databases for other similar sequences and reference multiple alignments of the full-length sequences were then constructed automatically using the PipeAlign protein family analysis toolkit [30]. The final output from the PipeAlign system is a high-quality, validated Multiple Alignment of Complete Sequences (MACS), in which the globular domains are accurately aligned. However, manual validation and refinement of the disordered regions is necessary, paying particular attention to the conserved LMs. An example alignment is shown in figure 2, corresponding to ELM entry MOD_PKB_1 and representing the motif recognized by protein kinase B (PKB/Akt/Rac-protein kinase) for serine/threonine phosphorylation.

In order to obtain information that might be used to improve the quality of multiple alignment programs, it is essential to provide sets of homologous sequences with different characteristics, representing specific problems for the alignment algorithm. One obvious issue is the similarity of the sequences to be aligned. It has been shown previously that multiple alignment methods can accurately align sequences that share similarity above the twilight zone of evolutionary relatedness, between 20–30% residue identity [41]. However, other characteristics may affect motif alignment quality, such as (i) sequence fragments, (ii) the presence of sequences containing false positive LMs that cannot be aligned on the motif instance or (iii) the presence of related sequences that do not contain the specific LM. In addition, alignment quality may depend on the nature of the LM itself: in terms of motif length, conservation and structural localisation. For these reasons, we have designed a number of different test sets that address these particular problems (see Methods for details). The number of alignments in each test set in shown in Table 1.

**Table 1:** BAliBASE reference 9 statistics. The number of alignments and the total number of sequences in the new Reference Set 9 of BAliBASE.

	Subset 1: TP sequences only			Subset 2: Sequences with errors	Subset 3: Sequences with FP motifs	Subset 4: Sequences with no motifs	Total
				
	V11 <20%	V12 20–40%	V13 40–80%				
No. of alignments	29	28	27	14	24	32	154
No. of sequences	423	228	263	490	985	1821	4210

The different Reference subsets were then used to analyse and compare eight different multiple alignment programs. In these tests, we concentrated on the accuracy of alignment of the LMs, rather than the quality of the overall alignment since this has been previously assessed in a number of different, independent studies [41,42].

### Accuracy of motif alignment at different levels of overall sequence similarity

The alignments in subset 1 consist of sequences that all contain the reference LM, and are divided into 3 different categories according to the residue similarity shared by the sequences (V11 contains sequences with <20% identity, V12 contains sequences with 20–40% identity and V13 contains sequences with 40–80% identity).

**Figure 3:**
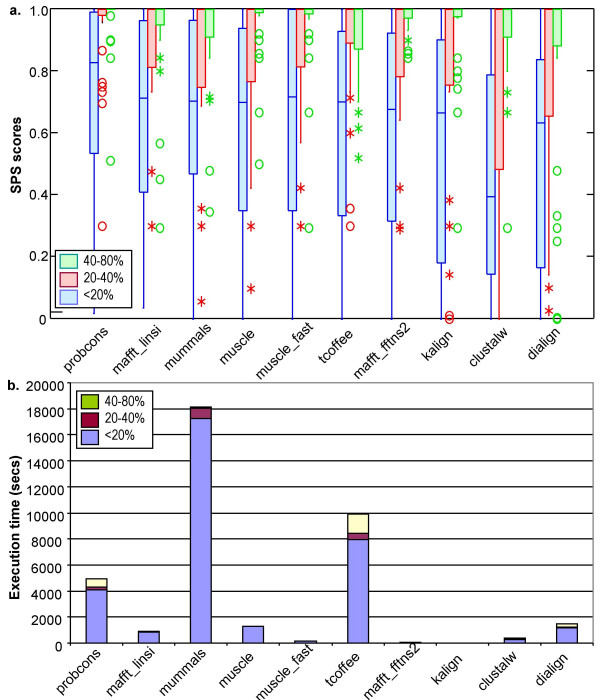
**Program SPS scores at different levels of overall sequence similarity**. a) Box plots of the SPS scores obtained by the different alignment programs in subset 1, showing the extreme observations (stars or circles), lower quartile, median, upper quartile, and largest observation in each similarity category. b) Execution times in seconds required to construct all the multiple alignments in Subset 1. Programs are displayed in the order of the Friedman test using the SPS scores for group V11 (additional file 1), with the highest scoring program on the left.

The first test is designed to study the effect of sequence similarity on motif alignment accuracy and is used as the basis for the subsequent tests. Figure 3a shows the SPS (sum-of-pairs scores) obtained by the different programs in the three similarity categories. A decrease in accuracy of alignment with decreasing overall sequence similarity is clearly observed for all the programs tested, with the greatest loss occurring in category V11 below 20% identity. Nevertheless, some alignment is still possible below this threshold, with most programs aligning more than 50% of the LMs successfully. The program Probcons achieves the highest scores in V11 with a mean of 73%, while Mafft_linsi (67%), Mummals (66%) and Muscle (65%) ranked next highest, although the differences are not statistically significant according to a Friedman rank sum test (additional file 1, table 1). The worst score at this level of identity was obtained by ClustalW, with a mean of 43%. The results of the Friedman test, together with a more detailed investigation of the scores obtained in category V11 (additional file 2, figure 1) show that none of the programs tested here scores systematically higher for all alignment test cases.

Although Probcons has improved the alignment of motifs in highly divergent sequences, it takes longer to run than MAFFT or MUSCLE. As shown in Figure 3b, the CPU time required to construct the complete set of alignments in Subset 1 is 900 sec for Mafft_linsi and 1297s for Muscle, compared to 4950 for Probcons. In a typical molecular biology study involving the analysis of a small number of sequences, the speed of the algorithm is generally not critical. However this may become an important deciding factor in high throughput experiments.

### Further investigation of factors affecting alignment quality

The similarity of the sequences is not the only factor affecting the accuracy of alignment of the LMs. As shown in figure 3a, the scores obtained by all the programs are highly variable (Inter Quartile Range from 0.44 for Probcons to 0.74 for Kalign), even when the sequences are in the same similarity range.

In order to further investigate possible causes for this variability, the LMs in subset 1, category V11, were annotated and classified according to three different criteria that might have a potential effect on motif alignment accuracy. Pearson correlation coefficients (shown in Table 2) were then calculated for the SPS quality scores obtained with the eight different alignment programs and (i) structural context (globular domain versus disordered region), (ii) motif conservation (at least 1 motif position fully conserved in alignment versus all variable positions) and (iii) motif length.

**Table 2:** Motif characteristics affecting quality of motif alignment.

	clustalw	dialign	kalign	mafft_linsi	mummals	muscle	probcons	tcoffee
context	**0.4601**	**0.5314**	**0.5686**	**0.6983**	**0.6030**	**0.6672**	**0.5528**	**0.6159**
conservation	0.1557	0.0848	-0.0070	0.0729	0.0707	-0.0037	0.0513	0.0370
length	0.1298	0.1857	0.0274	0.0147	0.0923	0.0324	0.0083	0.0339

Pearson correlation coefficients for SPS scores versus structural, motif conservation and motif length, for the eight different alignment programs evaluated. Values in bold indicate a significant correlation (critical value = 0.306 for 95% confidence interval)

**Figure 4:**
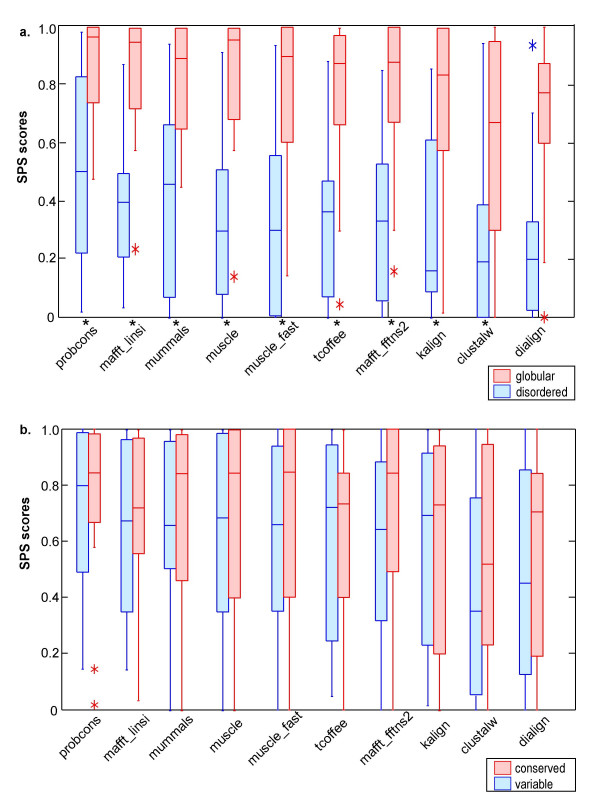
**Program SPS scores depending on different motif characteristics**. Box plots of the SPS scores obtained by the different alignment programs in subset 1, group V11 (<20% identity) under different conditions. The boxplots indicate the extreme observations (stars), lower quartile, median, upper quartile, and largest observation. Significant differences, according to a Wilcoxon signed ranks test (p < 0.05), are indicated by an asterix on the x-axis. P-values for the Wilcoxon tests are available in additional file 1, table 2. a) SPS scores for motifs found in globular domains versus disordered regions. b) SPS scores for motifs with a conserved residue versus variable motifs.

The largest correlation, for all the programs tested, is observed with the structural context of the LM (figure 4a). In this context, globular domains were predicted using the IUPred [30] program. In fact, the highest SPS scores are obtained for LMs that occur within globular domains (19 alignments) compared to the LMs found in disordered regions (11 alignments). Somewhat surprisingly, less correlation was observed with motif conservation (figure 4b). This is probably due to the very short, degenerate nature of most LMs, even those defined as 'conserved' (see Methods). Although conserved motifs are generally aligned better than variable ones, the differences are not significant (p = 0.05).

### Accuracy of motif alignment when other sequences are included in the alignment

**Figure 5:**
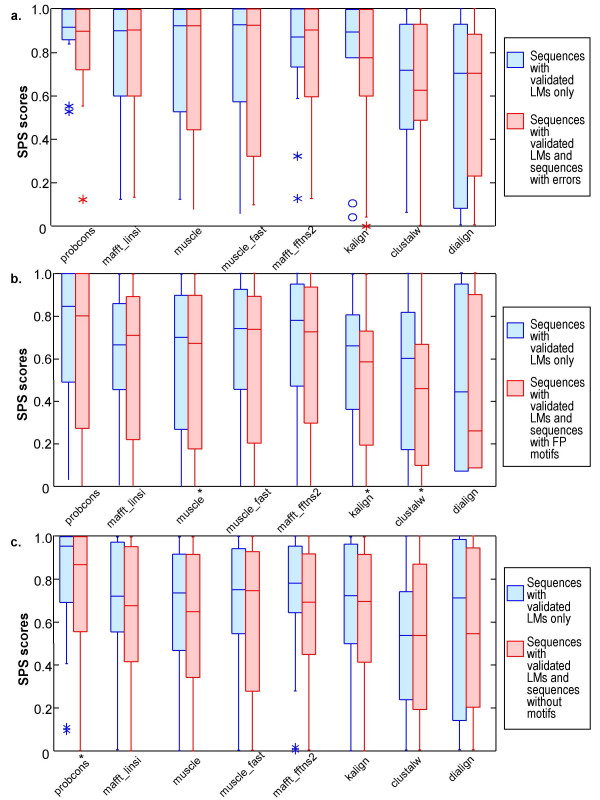
**Program SPS scores after inclusion of sequences without validated motifs**. Box plots of the SPS scores obtained by the different alignment programs under different conditions, showing the extreme observations (stars or circles), lower quartile, median, upper quartile, and largest observation. Significant differences, according to a Wilcoxon signed ranks test (p < 0.05), are indicated by an asterix on the x-axis. P-values for the Wilcoxon tests are available in additional file 1, table 3. a) SPS scores for alignments of sequences with validated motifs only compared to alignments including sequences with errors. b) SPS scores for alignments of sequences with validated motifs only compared to alignments including sequences containing false positive (FP) motifs. c) SPS scores for alignments of sequences with validated motifs only compared to alignments including sequences that do not contain any examples of the motif.

Here we test the degree to which the alignment of the validated LMs is disrupted by the introduction of other sequences. Figure 5 shows the SPS scores obtained by the different alignment programs, in the presence of a) sequences with errors, b) sequences with false positive motifs (sequences that have at least one domain in common with the query sequence and have a match to the ELM regular expression, but do not align in the region of the motif instance), c) sequences that have at least one domain in common with the query sequence, do not contain any matches the ELM regular expression and do not align in the region of the motif instance. The two slowest alignment methods, namely Mummals and Tcoffee were excluded from these tests, due to their excessive time and memory requirements.

In the case of sequences with 'errors', i.e. predicted sequences with missing exons, sequence fragments or splicing variants, the alignment of the validated motifs is not significantly affected for any of the programs tested. As might be expected, sequences with false positive motifs have a greater effect on alignment quality, and ClustalW, Kalign and Muscle show a significant reduction in alignment accuracy. More surprisingly, the introduction of related sequences that do not contain the LM also has an effect on alignment quality, although the difference is generally not significant.

### Quality of overall alignment

**Figure 6:**
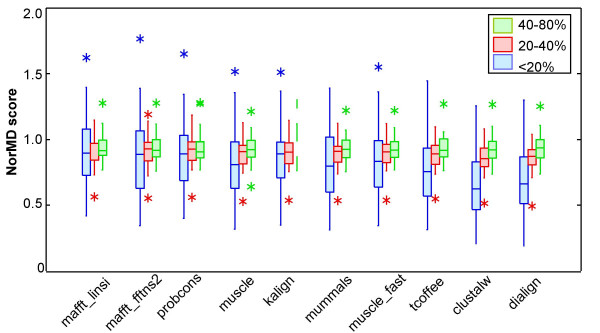
**Program NorMD scores at different levels of overall sequence similarity**. Box plots of the NorMD scores, representing the alignment quality of the full length alignment, obtained by the different alignment programs for the different similarity categories in subset 1. The boxplots indicate the extreme observations (stars), lower quartile, median, upper quartile, and largest observation. Programs are displayed in the order of the Friedman test using the NorMD scores for group V11 (additional file 1), with the highest scoring program on the left.

The SPS scores obtained in the tests described above represent the accuracy of the alignment of the validated LMs. They do not in any way indicate the overall quality of the complete alignment, and notably, they do not take into account the alignment of the homologous globular domains. Unfortunately, we do not have high quality reference alignments for the full length proteins in these tests. Therefore, in order to estimate the overall alignment quality, we calculated the NorMD scores [44] for the full-length sequences in the reference alignments in subset 1 (figure 6). NorMD is based on the Mean Distance (MD) scores introduced in ClustalX [33] and combines the advantages of a column-scoring technique with the sensitivity of methods incorporating residue similarity scores. The normalised scores allow us to define a cutoff above which the alignment is probably of high quality.

For sequences sharing >20% residue identity, all the programs tested achieved high quality alignments with mean NorMD scores above 0.8 confirming previous studies [30,31]. For the more divergent sequences in the twilight zone of sequence similarity (<20% identity), alignment quality is more variable (additional file 1, table 4), with the more recent methods, Mafft, Probcons, Muscle and Kalign, achieving the highest scores. A comparison of the NorMD scores (representing overall alignment quality) versus the SPS scores (representing LM alignment quality) (see additional file 2, figure 2) shows that LM alignment accuracy is not correlated with the overall quality of the complete alignment, which consists predominately of globular domains (Pearson correlation coefficient = 0.348; p < 0.05).

## Discussion

### The BAliBASE benchmarking suite

The systematic application of benchmarking approaches has been shown to be effective in many areas of bioinformatics, both for objective validation of existing tools and for identification of promising directions for future research. For example, the original version of the BAliBASE benchmark has been widely used to evaluate multiple sequence alignment methods and has lead to significant improvements in alignment quality [42,43]. The alignments in BAliBASE (version 1) were organised into Reference Sets that were specifically designed to represent the problems encountered by multiple alignment methods when aligning globular proteins, from a small number of divergent sequences, to sequences with large N/C-terminal extensions or internal insertions. In version 2 of the benchmark, three new Reference Sets were included, devoted to the particular problems posed by sequences with transmembrane regions, repeats and inverted domains.

The main objective of this study was to establish a benchmarking system that could be used to compare, evaluate and improve the alignment of short motifs in protein sequences. These linear motifs (LMs), including protein interaction sites, cell compartment targeting signals, post-translational modification sites and cleavage sites, play an essential role in the correct functioning of many multidomain proteins. The multiple alignments, developed here and included in the new BAliBASE Reference Set 9, represent real test cases and contain examples of experimentally verified functional motifs, extracted from the ELM database. In this version of the benchmark, we only included LMs that could be accurately aligned using a manual validation process. Motifs that could not be aligned unambiguously were removed from the reference alignments. This led to a bias in the benchmark test sets towards the known linear motifs that occur in globular domains, which is clearly not representative of all possible linear motifs, many of which are found in disordered regions that are difficult (or impossible) to align based solely on sequence information. In this case, the incorporation of multiple sequence alignment methods will not improve the motif prediction accuracy and other information will be needed, such as 3D structure, taxonomic range or cellular context. Nevertheless, it should be possible in future versions of the benchmark to include examples of these more difficult motifs, whenever experimental or other evidence allows an objective assessment of the alignment.

The number of sequences in each reference alignment was then augmented by identifying related proteins in the public databases containing putative functional sites. The alignment of these potential motifs was validated and refined manually to ensure the correct alignment of the conserved residues.

An important feature of the benchmark is the organisation of the reference alignments into different subsets that represent typical scenarios and problems that occur when trying to align short linear motifs in the context of a global multiple alignment. Thus, Subset 1 provides sequence sets at different levels of residue similarity, ranging from conserved sequences sharing 40–80% residue identity to more divergent sequences with <20% residue identity. In these alignments, all the sequences contain validated LMs. Subsets 2–4 provide alignments that represent a control to avoid over-optimisation of multiple alignment algorithms when using the Reference Set as a benchmark. For example, in Subset 3, aligning the false positive matches on the ELM motif instance would lead to a misalignment of the globular regions in the protein sequences. Subsets 2 and 4 consist of alignments that include sequences that do not contain the ELM motif instance and alignment of these sequences on the ELM motif instance would lead to over-prediction in subsequent motif discovery systems.

### Accuracy of motif alignment

The assessment of alignment quality was based only on the sequences containing verified motifs and only took into account the alignment of the LMs, ignoring the globular domain regions and the less well conserved, disordered regions. Nevertheless, we have shown that the accuracy of the LM alignment depends critically on the overall similarity of the sequence set, with a significant loss of accuracy for the most divergent sequences sharing less than 20% residue identity. This is true for all the programs tested in this study, regardless of whether a global or local alignment algorithm is used. For sequences sharing more than 20% identity, all the programs tested are capable of correctly aligning on average 80% of the motifs. Below this level of identity, in the "twilight zone" of protein sequences [41], the alignments produced by the programs are often unreliable with a larger dispersion of the scores. However, more recent programs, such as Mafft, Muscle, Mummals and Probcons, achieve significantly better scores here than the more traditional methods. Further investigation showed that the motif alignment quality for the most divergent sequences was significantly affected by the structural context of the motif. In fact, motifs that occurred within globular domains were aligned more successfully, compared to the motifs found in disordered regions. This result is not surprising since most multiple alignment programs are optimised for the alignment of globular domains. Introducing noise in the form of other related sequences, with or without false positive motifs, also affected the ability of some of the programs to correctly align the manually validated motifs. Currently, Probcons provides the best alignment of LMs (assuming the user does not attempt to modify the default parameters) and should therefore be used where quality is paramount and slow execution is not a hindrance. In the conservation score pipeline recently developed for the ELM resource [19], Probcons would be too slow, and therefore Mafft has been adopted, since it provides the best current combination of performance and motif alignment quality.

## Conclusion

Most of the modern multiple sequence alignment programs have not been optimised to align the full length, highly modular protein sequences which abound in the human and other eukaryotic proteomes. Instead, they have primarily been designed to align individual globular domains by reference to their 3D structure superpositions: i.e. relatively short and collinear sequence regions. We consider that there may be a number of different ways to improve alignment of motifs, by taking them into account either when developing new alignment algorithms and optimising parameters, or when performing the alignment. Hopefully, an in-depth investigation of the factors affecting LM alignment quality and a subsequent optimisation of the diverse parameters should improve the performance of the different alignment programs. In this case, the risk of overfitting to the motifs may be acceptable when motif detection is the prime consideration.

We have shown that none of the algorithms tested here is capable of reliably aligning all the LMs in distantly related sequences. It should be possible in the future to improve the reliability of LM alignment, either by combining a number of different algorithmic approaches and selecting a consensus alignment, or by including other contextual information, such as 3D structure, taxonomy or cellular context. If the resulting co-operative, knowledge based systems are capable of identifying the globular domain organisation of the proteins, then it should be possible to apply dedicated algorithms within the disordered, inter-domain regions, that can be used for the detection and alignment of over-represented or conserved sequence motifs. The success of such an approach will clearly hinge on the pertinence of the scoring function used to distinguish real motifs from random noise.

The use of more accurate multiple sequence alignments should in turn increase the precision of the new motif discovery systems now being developed, with subsequent consequences in a number of important applications. For example, since LMs represent sites of protein interaction and act as nodes in regulatory networks, cell signalling cannot be understood until we can accurately identify such functional motifs. Furthermore, linear motif targets are now considered to play a role in drug discovery [45], as exemplified by the nutlins, lead compounds that block the P53:MDM2 interaction with dramatic effects on cultured tumour cells [46,47].

## Competing interests

The authors declare that they have no competing interests.

## Authors' contributions

EP constructed the reference multiple alignments and contributed to their validation and analysis. CC conceived the initial idea together with JDT and TJG and contributed to the definition of the scoring methods. TJG and OP contributed to the formalization of the alignment program evaluation. JDT supervised and took part in all stages of the project. All authors read and approved the final manuscript.

## Supplementary Material

Additional File 1 Legend to additional figures S1, S2 and additional tables S1-4. Tables contain Friedman rank sum tests for data shown in figures 4 and 7 and Wilcoxon signed rank tests for data shown in figures 5 and 6 of the main article. Click here for file

Additional File 2 Figure S1. Comparison of SPS scores for LM alignment (y-axis) for each reference dataset (x-axis) in Subset 1, V11 (<20% identity). The scores obtained by the different programs are shown in different colours and the maximum score obtained for each reference dataset is indicated by a red circle. On the x-axis, **g **denotes LMs found in a globular domain, while **n **denotes LMs found in a non-globular domain. Figure S2. Comparison of motif alignment accuracy (SPS score) versus overall quality of complete alignment (NorMD score) obtained by the different alignment programs for the different similarity categories in subset 1 (blue = V1, <20% identity; red = V2, 20–40% identity; green = V3, 40–80% identity). Click here for file
