# Supplementary material for: A new protein linear motif benchmark for multiple sequence alignment software
Source: BMC Bioinformatics. 2008 Apr 25;9:213. doi: 10.1186/1471-2105-9-213 (PMC2374782; doi:10.1186/1471-2105-9-213)
Supplement: Additional File 1 — Legend to additional figures S1, S2 and additional tables S1-4. Tables contain Friedman rank sum tests for data shown in figures 4 and 7 and Wilcoxon signed rank tests for data shown in figures 5 and 6 of the main article. [file 1471-2105-9-213-S1.doc]

## Figure 1 - Comparison of motif alignment accuracy for a number of different programs

Comparison of SPS scores for LM alignment (y-axis) for each reference dataset (x-axis) in Subset 1, V11 (<20% identity). The scores obtained by the different programs are shown in different colours and the maximum score obtained for each reference dataset is indicated by a red circle. On the x-axis, **g** denotes LMs found in a globular domain, while **n** denotes LMs found in a non-globular domain.

## Figure 2 - Comparison of motif alignment accuracy versus overall quality of complete alignment

Comparison of SPS scores for LM alignment versus overall global alignment NorMD scores for all programs obtained in Subset 1. The different similarity categories in subset 1 are shown in different colours: V11 (<20% identity) – blue, V12 (20-40% identity) – red, V13 (40-80% identity) - green.

**Table 1 – Results of the Friedman rank test** (S=10, N=29, test statistic = 33.6, p=0.0001) to compare the performance of the programs based on SPS scores for V11 (<20% identity) shown in figure 4 in main article. For each test alignment, the programs are assigned a rank between 1 and 10 (with 10 indicating the highest scoring program). The ranks are then summed over all alignments. Thus, a higher rank sum indicates that a program tends to achieve higher scores. The programs are listed in rank sum order. An asterisk indicates that the two corresponding programs cannot be differentiated using the Friedman test (α= 5%).

| **Program** | **Rank sum** | **1** | **2** | **3** | **4** | **5** | **6** | **7** | **8** | **9** | **10** |
| --- | --- | --- | --- | --- | --- | --- | --- | --- | --- | --- | --- |
| **1. probcons** | 198.07 | ***** | ***** | ***** | ***** | ***** |  |  |  |  |  |
| **2. mafft_linsi** | 187.92 | ***** | ***** | ***** | ***** | ***** | ***** |  |  |  |  |
| **3. mummals** | 180.96 | ***** | ***** | ***** | ***** | ***** | ***** | ***** | ***** |  |  |
| **4. muscle** | 178.64 | ***** | ***** | ***** | ***** | ***** | ***** | ***** | ***** |  |  |
| **5. muscle_fast** | 158.92 | ***** | ***** | ***** | ***** | ***** | ***** | ***** | ***** | ***** | ***** |
| **6. tcoffee** | 156.02 |  | ***** | ***** | ***** | ***** | ***** | ***** | ***** | ***** | ***** |
| **7. mafft_fftns2** | 151.38 |  |  | ***** | ***** | ***** | ***** | ***** | ***** | ***** | ***** |
| **8. kalign** | 149.93 |  |  | ***** | ***** | ***** | ***** | ***** | ***** | ***** | ***** |
| **9. clustalw** | 117.45 |  |  |  |  | ***** | ***** | ***** | ***** | ***** | ***** |
| **10. dialign** | 115.42 |  |  |  |  | ***** | ***** | ***** | ***** | ***** | ***** |

**Table 2 – Wilcoxon signed ranks test** for comparison of SPS scores for V11 (<20% identity) shown in figure 5 in main article.

| **SPS for alignments with motifs found in globular domains versus disordered regions:** | | | | | | | | | |
| --- | --- | --- | --- | --- | --- | --- | --- | --- | --- |
| clustalw | dialign | kalign | mafft_fftns2 | mafft_linsi | mummals | muscle_fast | muscle | probcons | tcoffee |
| 0.0415 | 0.0615 | 0.0122 | 0.0122 | 0.0034 | 0.0210 | 0.0161 | 0.0122 | 0.0068 | 0.0093 |
| **SPS for alignments with motifs with a conserved residue versus variable motifs:** | | | | | | | | | |
| clustalw | dialign | kalign | mafft_fftns2 | mafft_linsi | mummals | muscle_fast | muscle | probcons | tcoffee |
| 0.2119 | 0.311 | 0.7651 | 0.4463 | 0.6333 | 0.3848 | 0.5452 | 0.6499 | 0.5750 | 0.7726 |

**Table 3 – Wilcoxon signed ranks test** for comparison of SPS scores obtained by the different alignment programs for subsets 2,3 and 4, shown in figure 6 in main article.

| **SPS for alignments with and without sequences containing errors:** | | | | | | | | | | | | | | |
| --- | --- | --- | --- | --- | --- | --- | --- | --- | --- | --- | --- | --- | --- | --- |
| clustalw | | dialign | | kalign | | mafft_fftns2 | | mafft_linsi | | muscle_fast | | muscle | | probcons |
| 0.3765 | | 0.4375 | | 0.0625 | | 0.3125 | | 0.5313 | | 0.0625 | | 0.6563 | | 0.3447 |
| **SPS for alignments with and without sequences with false positive motifs:** | | | | | | | | | | | | | | |
| clustalw | | dialign | | kalign | | mafft_fftns2 | | mafft_linsi | | muscle_fast | | muscle | | probcons |
| 0.0461 | | 0.1484 | | 0.0078 | | 0.0820 | | 0.1613 | | 0.0244 | | 0.0151 | | 0.1167 |
| **SPS for alignments with and without sequences with false negative sequences:** | | | | | | | | | | | | | | |
| clustalw | dialign | | kalign | | Mafft_fftns2 | | mafft_linsi | | muscle_fast | | muscle | | probcons | |
| 0.3659 | 0.2276 | | 0.1090 | | 0.0895 | | 0.0876 | | 0.1768 | | 0.2653 | | 0.0474 | |

**Table 4 –** Results of the Friedman rank test (S=10, N=29, test statistic = 148.1, p=0.0) to compare the performance of the programs based on NorMD scores for V11 (<20% identity) shown in figure 7 in main article. For each test alignment, the programs are assigned a rank between 1 and 10 (with 10 indicating the highest scoring program). The ranks are then summed over all alignments. Thus, a higher rank sum indicates that a program tends to achieve higher scores. The programs are listed in rank sum order. An asterisk indicates that the two corresponding programs cannot be differentiated using the Friedman test (α= 5%).

| **Program** | **Rank sum** | **1** | **2** | **3** | **4** | **5** | **6** | **7** | **8** | **9** | **10** |
| --- | --- | --- | --- | --- | --- | --- | --- | --- | --- | --- | --- |
| **1. mafft_linsi** | 255.95 | ***** |  |  |  |  |  |  |  |  |  |
| **2. mafft_fftns2** | 218.08 |  | ***** | ***** | ***** |  |  |  |  |  |  |
| **3. probcons** | 205.03 |  | ***** | ***** | ***** | ***** |  |  |  |  |  |
| **4. muscle** | 196,91 |  | ***** | ***** | ***** | ***** |  |  |  |  |  |
| **5. kalign** | 185.02 |  |  | ***** | ***** | ***** |  |  |  |  |  |
| **6. mummals** | 147.03 |  |  |  |  |  | ***** | ***** |  |  |  |
| **7. muscle_fast** | 144.13 |  |  |  |  |  | ***** | ***** |  |  |  |
| **8. tcoffee** | 118.90 |  |  |  |  |  |  |  | ***** |  |  |
| **9. clustalw** | 63.51 |  |  |  |  |  |  |  |  | ***** | ***** |
| **10. dialign** | 57.13 |  |  |  |  |  |  |  |  | ***** | ***** |
