# Supplementary material for: A new protein linear motif benchmark for multiple sequence alignment software
Source: BMC Bioinformatics. 2008 Apr 25;9:213. doi: 10.1186/1471-2105-9-213 (PMC2374782; doi:10.1186/1471-2105-9-213)
Supplement: Additional File 2 — Figure S1. Comparison of SPS scores for LM alignment (y-axis) for each reference dataset (x-axis) in Subset 1, V11 (<20% identity). The scores obtained by the different programs are shown in different colours and the maximum score obtained for each reference dataset is indicated by a red circle. On the x-axis, g denotes LMs found in a globular domain, while n denotes LMs found in a non-globular domain. Figure S2. Comparison of motif alignment accuracy (SPS score) versus overall quality of complete alignment (NorMD score) obtained by the different alignment programs for the different similarity categories in subset 1 (blue = V1, <20% identity; red = V2, 20–40% identity; green = V3, 40–80% identity). [file 1471-2105-9-213-S2.ppt]

## Slide 1
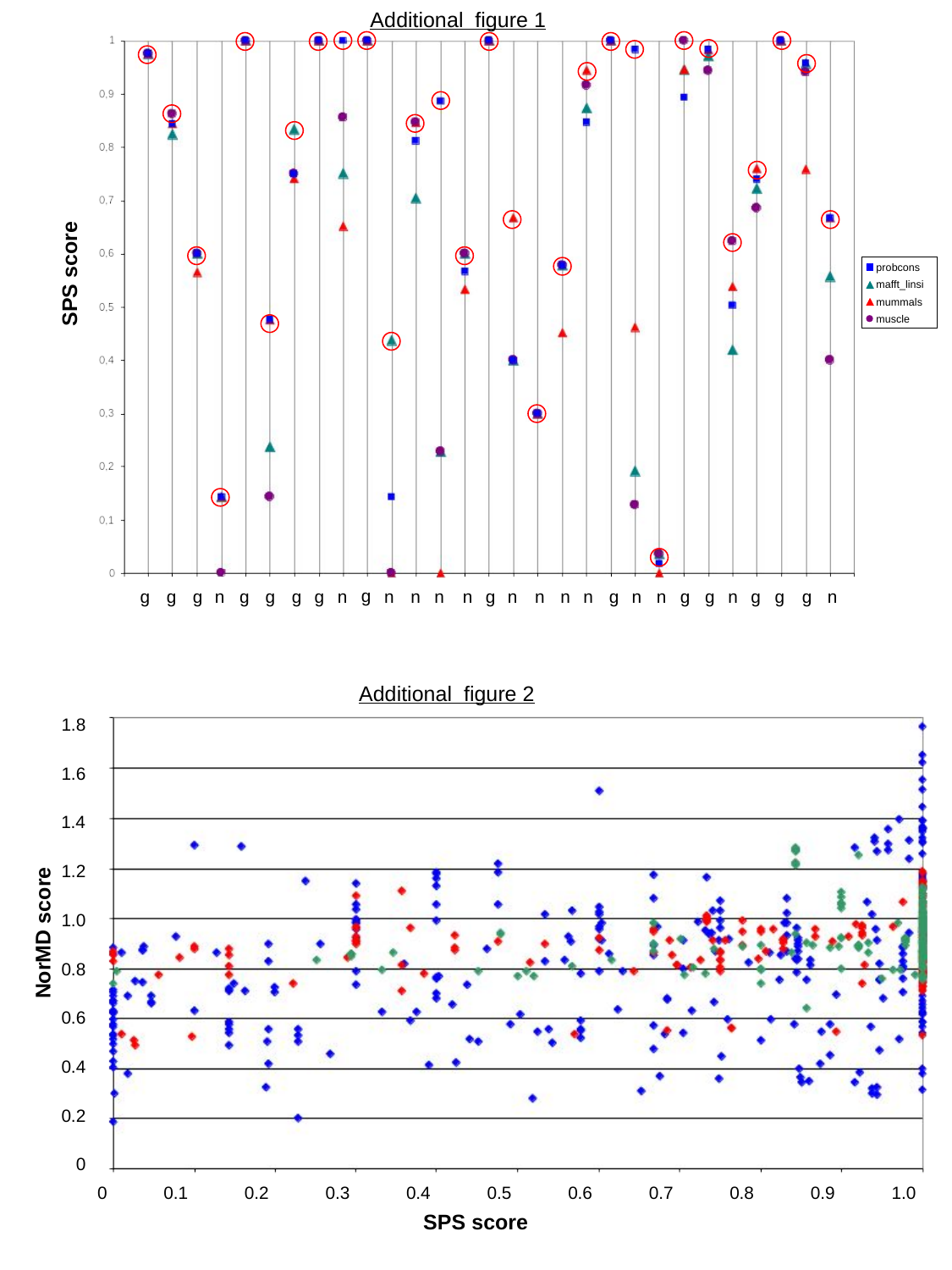

Additional figure 1
SPS score
probcons
mafft_linsi
mummals
muscle
g
n
n
g
g
g
n
g
g
g
g
n
n
n
n
g
n
n
n
g
n
n
g
g
n
g
g
g
n
Additional figure 2
1.8
1.6
1.4
1.2
1.0
NorMD score
0.8
0.6
0.4
0.2
0
0
0.1
0.2
0.3
0.4
0.5
0.6
0.7
0.8
0.9
1.0
SPS score
